# Laboratory Characteristics on SARS-CoV-2 Infection among Patients with Diabetes Mellitus: A Single-Center Retrospective Study

**DOI:** 10.1155/2023/9940250

**Published:** 2023-01-18

**Authors:** Raneem O. Salem, Ayesha Nuzhat, Shawana Zaheer, Majd Aldeen Kallash

**Affiliations:** ^1^Basic Medical Sciences Department, King Fahad Medical City, King Saud Bin Abdul-Aziz University of Health Sciences, Riyadh, Saudi Arabia; ^2^Diabetic Center and Endocrinology, King Fahad Medical City, Riyadh, Saudi Arabia

## Abstract

**Background:**

Diabetic patients have been severely affected by COVID-19 infection. It has been reported that the disease is more progressive leading to venous and arterial thromboembolism, due to multiple factors. This study was conducted to determine the hematologic parameters including D-dimer in diabetic patients with COVID-19 infection in association with disease severity and treatment.

**Method:**

This retrospective cohort study was conducted at King Fahad Medical City, Saudi Arabia, after obtaining IRB approval, by collecting data regarding all laboratory parameters, disease severity, and anticoagulant treatment of COVID-19 diabetic patients (*n* = 159) from medical records from March to December 2020.

**Result:**

Mean value of white blood cells, neutrophils, monocytes, eosinophils, lymphocyte monocyte ratio, C-reactive protein, serum ferritin, and LDH levels was elevated in severe cases than in mild cases with statistical significant increase in HbA1c (0.047), serum fibrinogen (0.007), C-reactive protein (0.005), serum ferritin (0.034), and serum LDH (0.015). Mortality was observed in 14 (8.8%) patients mostly with severe COVID-19 with diabetes. In our study, treatment with low molecular weight heparin was not significantly related to severity. A logistic regression analysis indicated an association of some laboratory parameters with severity and mortality of the disease.

**Conclusion:**

The routine blood parameters if detected early will enable physicians to identify severe cases of COVID-19 patients with Diabetes for prompt treatment and save considerable time and resources.

## 1. Introduction

The COVID-19 has already infected more than 643 million people and resulted in more than 6.62 million deaths globally [1]. The average of country-/territory-specific COVID-19 CFR is about 2%–3% worldwide [2]. COVID-19 has varied clinical manifestations from trivial flu-like symptoms to multiple system failure and death, especially due to significant impact on the hematopoietic system and hemostasis.

It has been reported that prevalence of diabetes was 20% among COVID-19 patients [3]. Disease severity and outcome in these patients are related to numerous markers like serum ferritin, C-reactive protein, interleukin-6, fibrinogen, serum LDH, and D-dimer. Hematological aberrations in COVID-19 are associated with disease progression, severity, and mortality. Lymphopenia, thrombocytopenia, and abnormal coagulation profile is very well reported in patients of COVID-19 [4–7]. In patients with COVID-19 infection, the disease is more severe leading to venous and arterial thromboembolism, due to multiple factors such as inflammation, hypoxia, immobilization, diabetes, and disseminated intravascular coagulation, and is associated with increased morbidity [8].

Li et al. reported that the mortality rate for diabetic patients with COVID-19 was 14.5% which is higher than nondiabetic patients (5.7%). This could be due to a higher level of D-dimer and low lymphocyte count at admittance. So, patients with COVID-19 and diabetes need extra attention [9]. Every patient with COVID-19 is required to be placed on prophylactic doses of anticoagulation, usually with low molecular weight heparin (LMWH), unless contraindicated [10, 11].

Kurt et al. reported that the leucocyte count, neutrophil count, neutrophil-to-lymphocyte ratio (NLR), platelet-to-lymphocyte ratio (PLR), and ferritin level were significantly higher in the severe disease subgroup than in the nonsevere subgroup (*p* < 0.001). The lymphocyte counts and lymphocyte-to-monocyte ratio (LMR) were significantly lower in the severe disease subgroup than in the nonsevere subgroup (*p* < 0.001). NLR was positively correlated with the COVID-19 risk (adjusted OR 1.438, *p* = 0.012) whereas the association of PLR and LMR with COVID-19 risk was unclear [12]. However, there are very few studies on specific information regarding hematological parameters of patients with diabetes and COVID-19.

These parameters if detected early can play a vital role in the early prediction of disease severity and can provide a better guide for prompt management of patients, thus helping in decreasing the disease morbidity and mortality.

### 1.1. Objective

This study was conducted to determine laboratory parameters including D-dimer in diabetic patients with COVID-19 infection in association with disease severity and treatment.

## 2. Method

### 2.1. Ethics Statement

This retrospective cohort study was conducted at King Fahad Medical City, Saudi Arabia, after obtaining IRB approval (IRB no: 20:737) on 29^th^ Nov 2020 from its IRB committee.

### 2.2. Case Definition (for SARS-CoV-2 Infection and for Diabetes Mellitus)

1. A person with a positive PCR test for COVID-19 with or without symptoms
2. Diabetes mellitus characterized by hyperglycemia (*HbA*1*c* > 6.5%) as indicated in the medical records

### 2.3. Data Collection and Participants

The participants were all patients at King Fahad Medical City with COVID-19 and diabetes. All laboratory parameters, disease severity, and anticoagulant treatment of COVID-19 diabetes were collected from patients (*n* = 209) from medical records between March and December 2020.

All laboratory data were collected at admission such as HbA1c, hemoglobin, total WBC count, differential count, platelets, neutrophil-to-lymphocyte ratio (NLR), lymphocyte-to-monocyte ratio (LMR), platelet-to-lymphocyte ratio (PLR), prothrombin time, activated PTT, fibrinogen, D-dimer, C-reactive protein, serum ferritin, and LDH.

### 2.4. Inclusion Criteria

All diabetic patients with COVID-19 infection >18 years were included.

### 2.5. Exclusion Criteria

Patients with incomplete records and prior hematological disorders were excluded.

**Figure 1:**
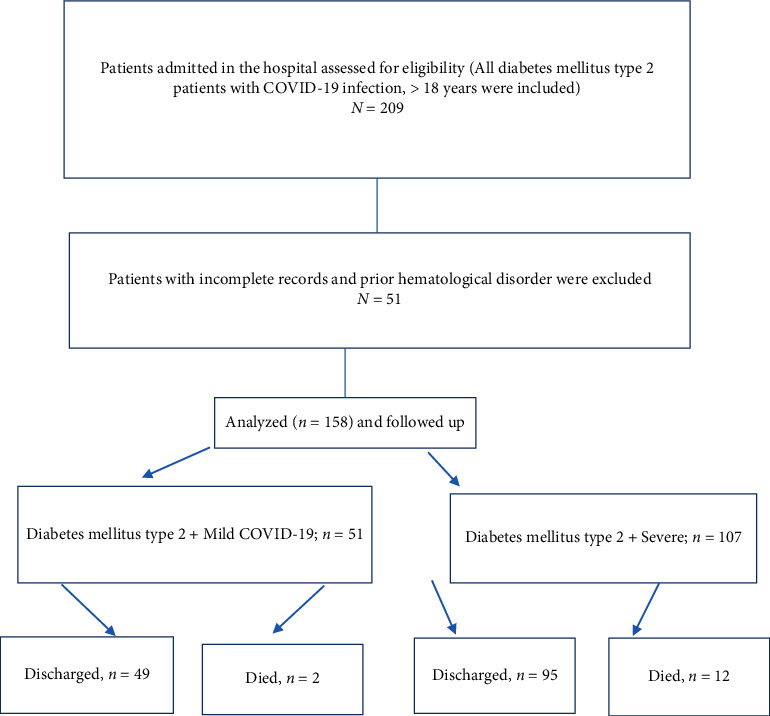
Recruitment and assessment flowchart for patients.

Patients were classified at the time of admission into mild and severe cases based on hospital guidelines at KFMC (Figure 1, flowchart).

Mild disease of COVID-19 was demarcated for patients with symptoms of fever, sore throat, cough, and no sign of pneumonia on X-rays. Patients with respiratory distress (respiratory rate > 30 breath per min, O_2_ saturation less than 93%, and more than 50% lung infiltrate) were classified as having severe disease [13].

### 2.6. Data Entry and Analysis

Data was transferred to excel and analyzed in SPSS version 22, Illinois, USA. Descriptive statistics were calculated for hematologic parameters and Pearson correlation to correlate these parameters with the severity of the disease. Laboratory parameters were compared between mild and severe cases using independent *t* tests. Quantitative variables such as hemoglobin, white blood count (WBC), and differential white blood count including neutrophil-to-lymphocyte ratio (NLR), lymphocyte-to-monocyte ratio (LMR), platelet-to-lymphocyte ratio (PLR), prothrombin time PT, activated partial thromboplastin time (APTT), ferritin, D-dimer, lactate dehydrogenase (LDH), and C-reactive protein (CRP) were analyzed. *p* value equal or less than 0.05 was considered significant. Multinomial logistic regression was conducted to know the impact of the lab parameters mentioned above on COVID-19 severity and mortality.

## 3. Results

209 patients were identified according to our inclusion and exclusion criteria. Patients with missing data or hematological disease were excluded (*n* = 51) (Figure 1). Demographic features and laboratory parameters of patients with COVID-19 infection and diabetes mellitus (*n* = 158) at the time of admission with mild or severe illness are depicted in Tables 1, 2, and 3.

Out of 158 patients, 94.3% of patients were more than 40 years, and COVID-19 with diabetes was predominant in males (91 (57.2%)) than females (68 (42.8%)). Severe cases were 107 (67.3%) and mild cases were 51 (32.1%). All demographic data were nonsignificant between mild and severe groups except for hospital stay which was significantly longer in severe cases (Table 1).

**Figure 2:**
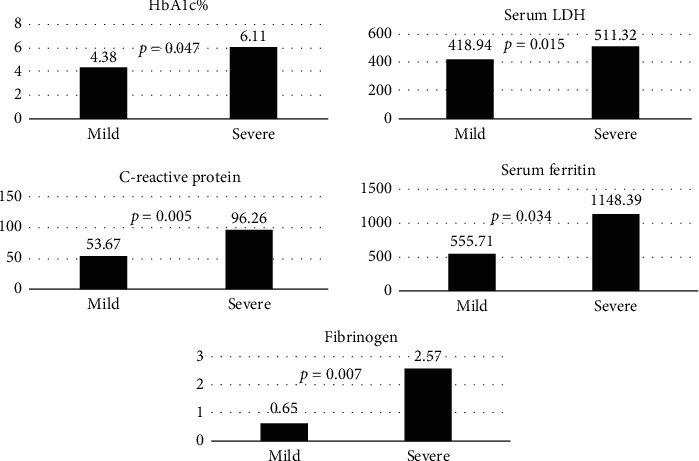
Correlation between COVID-19 severity and lab parameters with clinical significance < 0.05.

The mean value of white blood cells, neutrophils, monocytes, eosinophils, lymphocyte-to-monocyte Ratio, C-reactive protein, serum ferritin, and LDH levels was elevated in severe cases than in mild cases with statistical significant increase in HbA1c (0.047), serum fibrinogen (0.007), C-reactive protein (0.005), serum ferritin (0.034), and serum LDH (0.015) (Table 3 and Figure 2).

Mortality was observed in 14 (8.8%) patients, and all of these had severe COVID-19 with diabetes. LMWH was started in 92.5 (%) of all cases.

We performed logistic regression and obtained the odds ratio for many factors. There was association between HbA1c (OR 1.129), hemoglobin (OR 1.026), neutrophils (OR 1.136), lymphocyte (OR 1.085), monocyte (OR 1.025), eosinophils (OR 2.017), PLR (OR 1.062), PT (OR 1.035), serum fibrinogen (OR 1.50), D-dimer (OR 1.03), C-reactive protein (OR 1.007), serum ferritin (1.000), and serum LDH (1.002) and severity of COVID-19 (Table 4).

In addition, there was association between certain blood parameters (hemoglobin (OR 1.026), WBC (OR 1.49), neutrophils (OR 2.34), lymphocyte (OR 2.42), monocyte (OR 3.61), eosinophils (OR 6.12), basophils (OR 1.00), PLR(OR 2.24), D-dimer (OR 2.307), C-reactive protein (OR 1.02), and serum ferritin (1.000)) and mortality of COVID-19 (Table 5). Eosinophil levels showed the strongest association with severity and mortality of the disease.

## 4. Discussion

Variations in hematological characteristics in patients with COVID-19 are evolving as imperative features of COVID-19 infection trigger coagulation activation, inflammatory reactions, hypercoagulability induction, and cytokine storms that ultimately indicate disease severity. The patient deterioration condition will lead to admission to the intensive care unit and death [14].

An analysis by Varikasuvu et al. showed that diabetic COVID-19 patients are highly vulnerable to coagulation abnormalities and inflammation than the nondiabetic COVID-19 cases. The levels of serum ferritin (standardized mean difference (SMD): 0.47), C-reactive protein (SMD = 0.53), interleukin-6 (SMD = 0.3), fibrinogen (SMD = 0.31), and D-dimers (SMD = 0.54) were increased in diabetic COVID-19 cases compared to nondiabetic COVID-19 patients, signifying coagulation irregularities and inflammatory storm [15]. Li conducted a study for 199 COVID-19 patients, 76 were diabetic and 123 were nondiabetic, and reported that the mortality rate of COVID-19 patients with diabetes was significantly higher than those without diabetes. Diabetes, higher level of D-dimer, and lymphocyte count less than 0.6 × 10^9^/L at admission were the risk factors associated with in-hospital death. Thus, diabetic patients are more prone to develop severe COVID-19 infection and higher mortality rate due to their hypercoagulable proinflammatory stage [9, 16].

In a retrospective cohort study of 380 patients, the numbers of lymphocytes and eosinophils were considerably lower in patients with critical disease than in those with severe or moderate disease (*p* < 0.0001), and prothrombin time, D-dimer, and fibrin degradation products considerably increased in severe cases (*p* < 0.0001). Thrombotic and hemorrhagic events played a major role in patient's death (19 [35%] of 55) [17].

Bansal et al. showed the significant association of D-dimers with the severity of the COVID-19 disease. Patients with acute lung injury and severe infection will have disseminated intravascular coagulation (DIC) secondary to sepsis [18]. Ayusha et al. reported that 1.5 *μ*g/mL is the optimal cutoff value of admission D-dimer for predicting mortality in COVID-19 patients, with good sensitivity and specificity suggesting that laboratory interpreters of clinical decline can aid in intensifying the care of the patients with this infection and support in suitable triaging and resource deployment [19]. In our study, it was more than 2.5 mg/L in both mild and severe cases.

A local study also reported that diabetic COVID-19 patients have a higher mortality rate than their nondiabetic counterparts, and patients with uncontrolled diabetes (HbA1C > 7%) on admittance often end up in intensive care [20]. In our study, mortality was observed in 14 (8.8%) patients, and all of these had severe COVID-19 with diabetes.

In our study, LMWH was started in 92.5% of all cases. Terpos et al. in a Chinese study reported that LMWH administration among patients with markedly elevated D-dimers or in those meeting the criteria for sepsis-induced DIC was significantly associated with improved 28-day overall survival, *p* = 0.017 and *p* = 0.029 among two patient groups, users versus nonusers, respectively [21].

The anti-inflammatory properties of LMWH may be beneficial in COVID-19 patients and should be integrated with other antithrombotic treatments such as antithrombin, and recombinant thrombomodulin to hinder the complex “immunothrombosis” process [10]. In our study, treatment with low molecular weight heparin was not significantly related to severity.

The association between laboratory parameters and severity of disease has been reported by other studies. Taj et al. in their study concluded that leukocytosis, neutrophilia, elevated neutrophil-to-lymphocyte ratio, APTT, D-dimer, LDH, serum ferritin, and CRP are associated with severity of COVID-19 disease. These laboratory biomarkers therefore play a vital role in the early prediction of the disease [7].

Many infections can be detected using the total blood count test, which can easily be available and cheap but at the same time very informative. Hematological, inflammatory, and other markers can be used to evaluate the severity and prognosis of COVID-19 diseases as there is predictive relevance because all SARS-CoV-2 genetic variants have a significant potential for thrombosis, especially in diabetic patients [22].

The addition of these parameters in the regimens for patients' categorization on admission will facilitate prompt effective intervention and proper decision making in the course of clinical case management [23].

## 5. Conclusion

In conclusion, COVID-19 disease affects the hematopoietic system in COVID-19 patients with diabetes leading to major complications and untimely death. This study determines laboratory parameters including D-dimer at admission in diabetic patients with COVID-19 infection in association with disease severity and mortality.

Further, optimal anticoagulation regimen with or without adjunct antithrombotic therapies such as LMWH may be useful in patients with COVID-19.

### 5.1. Limitations

Our study has certain limitations. Some patients were excluded because of incomplete documentation of laboratory testing. Our study design is retrospective; hence, findings are limited to the accuracy of record keeping. Furthermore, duration of DM was not recorded which may affect the findings. The results should be interpreted with the caution that diabetes may coexist with other conditions such as cardiac diseases in COVID-19 patients. Therefore, further well-controlled studies are needed in the future to establish an independent role of diabetes in COVID-19.

These limitations do not undervalue the findings of the present study which are robust and add value to the limited literature on COVID-19 patients within the Arab region. Further, lack of a control group is another limitation. We suggest multicenter studies to clarify the role of lab parameters in the severity of disease and mortality in COVID-19.

## Figures and Tables

**Table 1:** Demographic features of patients with COVID-19 infection and diabetes mellitus at the time of admission with mild or severe illness (*n* = 158).

General characteristics	Mild *N* = 51	Severe *N* = 107	*p* value
	*N* (%)	*N* (%)	
Ethnicity			
Saudi	36 (22.8%)	69 (43.7%)	0.448
Non-Saudi	15 (9.5%)	38 (24.1%)	
Gender			
Male	29 (18.4%)	61 (38.6%)	0.986
Female	22 (13.9%)	46 (29.1%)	
Age			
<40	4 (2.5%)	5 (3.2%)	0.421
>40	47 (29.7%)	102 (64.6%)	
Mortality			
Yes	2 (1.3%)	12 (7.6%)	0.133
No	49 (31%)	95 (60.1%)	
Length of hospital stay			
<3 weeks	40 (78.4%)	93 (86.92%)	0.049^∗^
>3 weeks	11 (21.6%)	14 (13.8%)	
ICU admission			
Yes	11 (7%)	30 (19%)	0.227
No	40 (25.3%)	77 (48.7%)	
LMWH given			
Yes	49 (31.4%)	97 (62.2%)	0.273
No	2 (1.3%)	8 (5.1%)	
Comorbidities			
Yes	41 (25.9%)	95 (60.1%)	0.154
No	10 (6.3%)	12 (7.6%)	
Complications during hospitalization			
Yes	4 (2.5%)	8 (5.1%)	0.935
No	47 (29.7%)	99 (62.7%)	

^∗^ *p*< 0.05.

**Table 2:** Hematological parameters of all patients with COVID-19 infection and diabetes mellitus at the time of admission.

Hematological parameter	All patients mean ± SD	Reference range
HbA1C at admission	5.56 (4.999)	4-5.6%
Hemoglobin (g/dL)	12.94 (2.251)	>13 in males >12 in females
WBC (10^9^ cells/L)	8.71 (7.676)	4.5-11
Neutrophil count (10^9^ cells/L)	71.58 (15.096)	2.0-8.0
Lymphocyte count (10^9^ cells/L)	19.41 (11.779)	1.0-4.8
Monocyte count (10^9^ cells/L)	7.72 (12.515)	0.30-0.90
Eosinophil count (10^9^ cells/L)	0.78 (1.93)	0-0.5
Basophil count (10^9^ cells/L)	0.30 (0.282)	0.02-0.05
LMR	3.63 (5.965)	2.97-4.83
PLR	21.86 (2.251)	36.63-172.68
NLR	6.38 (7.970)	0.3-2.1
Platelet count (10^9^ cells/L)	259.50 (97.366)	150-400
Activated PTT (secs)	31.52 (4.349)	30-40
PT (secs)	13.62 (2.285)	11-13.5
Fibrinogen (g/L)	1.86 (3.710)	2.0-4.0
D-Dimer (mg/L)	2.73 (8.940)	<0.50
C-reactive protein (mg/L)	81.67 (87.06)	<10
Serum ferritin (*μ*g/L)	954.70 (162.72)	12-300
LDH (units/L)	481.13 (221.946)	140-280

LMR: lymphocyte/monocyte ratio; PLR: platelet/lymphocyte ratio; NLR: neutrophil/lymphocyte ratio.

**Table 3:** Hematological parameters of mild and severe patients with COVID-19 infection and diabetes mellitus at the time of admission.

Hematological parameter	Mild cases	Severe cases	Reference range	*p* value
HbA1C at admission	4.38 (5.043)	6.11 (4.929)	4-5.6%	0.047^∗^
Hemoglobin (g/dL)	12.74 (2.352)	13.06 (2.195)	>13 in males >12 in females	0.399
WBC (10^9^ cells/L)	8.66 (5.677)	8.75 (8.518)	4.5-11	0.944
Neutrophil count (10^9^ cells/L)	70.66 (15.717)	72.02 (14.848)	2.0-8.0	0.604
Lymphocyte count (10^9^ cells/L)	20.25 (12.932)	19.00 (11.225)	1.0-4.8	0.540
Monocyte count (10^9^ cells/L)	7.16 (4.237)	8.00 (14.963)	0.30-0.90	0.699
Eosinophil count (10^9^ cells/L)	0.55 (1.053)	0.90 (2.229)	0-0.5	0.306
Basophil count (10^9^ cells/L)	0.33 (0.321)	0.29 (.262)	0.02-0.05	0.364
LMR	3.35 (2.846)	3.80 (7.002)	2.97-4.83	0.659
PLR	25.20 (34.648)	20.47 (24.956)	36.63-172.68	0.330
NLR	7.02 (8.712)	6.13 (7.631)	0.3-2.1	0.513
Platelet count (10^9^ cells/L)	266.53 (105.14)	255.83 (94.164)	150-400	0.521
Activated PTT (secs)	31.26 (4.159)	31.61 (4.462)	30-40	0.639
PT (secs)	13.42 (1.855)	13.71 (2.475)	11-13.5	0.446
Fibrinogen (g/L)	0.65 (1.621)	2.57 (4.358)	2.0-4.0	0.007^∗^
D-Dimer (mg/L)	3.28 (10.687)	2.45 (7.959)	<0.50	0.593
C-reactive protein (mg/L)	53.67 (68.892)	96.26 (92.177)	<10	0.005^∗^
Serum ferritin (*μ*g/L)	555.71 (716.488)	1148.39 (1885.9)	12-300	0.034^∗^
LDH (units/L)	418.94 (251.46)	511.32 (200.538)	140-280	0.015^∗^

^∗^ *p* value < 0.05.

**Table 4:** Logistic regression analysis to know the impact of lab parameters on COVID-19 severity.

Hematological parameter	Parameter estimate (PE)	SE	*p* value	Odds ratio (*B*)	Confidence interval (95%)	
					Lower	Upper
HbA1C at admission	0.122	0.061	0.047^∗^	1.129	1.001	1.222
Hemoglobin (g/dL)	0.026	0.135	0.850	1.026	0.787	1.337
WBC (10^9^ cells/L)	0.132	0.107	0.216	0.876	0.711	1.080
Neutrophil count (10^9^ cells/L)	0.127	0.093	0.171	1.136	0.946	1.363
Lymphocyte count (10^9^ cells/L)	0.081	0.104	0.432	1.085	0.885	1.329
Monocyte count (10^9^ cells/L)	0.025	0.044	0.566	1.025	0.941	1.117
Eosinophil count (10^9^ cells/L)	0.701	0.271	0.010^∗^	2.017	1.18	3.430
Basophil count (10^9^ cells/L)	1.68	1.30	0.197	0.186	0.015	2.389
NLR	0.291	0.166	0.081	0.748	0.540	1.036
LMR	0.130	0.149	0.382	0.878	0.656	1.175
PLR	0.060	0.056	0.287	1.062	0.951	1.186
Platelet count (10^9^ cells/L)	0.006	0.006	0.306	0.994	0.984	1.005
Activated PTT (secs)	0.092	0.078	0.237	0.912	0.783	1.062
PT (secs)	0.126	0.131	0.335	1.135	0.878	1.467
Fibrinogen (g/L)	0.405	0.203	0.046^∗^	1.500	1.008	2.232
D-Dimer (mg/L)	0.030	0.035	0.398	1.030	0.961	1.104
C-reactive protein (mg/L)	0.007	0.004	0.104	1.007	0.999	1.016
Serum ferritin (*μ*g/L)	0.000	0.000	0.375	1.000	1.000	1.001
LDH (units/L)	0.002	0.001	0.216	1.002	0.999	1.005

^∗^ *p* value < 0.05.

**Table 5:** Logistic regression analysis to know the impact of lab parameters on COVID-19 mortality.

Hematological parameter	Parameter estimate (PE)	SE	*p* value	Odds ratio (*B*)	Confidence interval (95%)	
					Lower	Upper
HbA1C at admission	0.146	0.171	0.394	0.864	0.618	1.209
Hemoglobin (g/dL)	0.786	0.586	0.180	2.19	0.695	6.925
WBC (10^9^ cells/L)	0.402	0.417	0.335	1.49	0.661	3.381
Neutrophil count (10^9^ cells/L)	16.97	17.04	0.320	2.34	0.000	7.626
Lymphocyte count (10^9^ cells/L)	17.00	17.07	0.319	2.42	0.000	8.241
Monocyte count (10^9^ cells/L)	12.79	15.30	0.403	3.61	0.000	3.833
Eosinophil count (10^9^ cells/L)	15.62	16.10	0.332	6.12	0.000	3.154
Basophil count (10^9^ cells/L)	25.33	21.24	0.233	1.00	0.000	1.219
NLR	6.54	3.67	0.075	0.001	0.000	1.916
LMR	9.47	5.93	0.111	0.000	0.000	8.746
PLR	0.811	0.427	0.058^∗^	2.24	0.974	5.196
Platelet count (10^9^ cells/L)	0.065	0.033	0.047^∗^	0.937	0.879	0.999
Activated PTT (secs)	0.065	0.170	0.702	0.937	0.671	1.308
PT (secs)	0.602	0.607	0.322	0.548	0.167	1.801
Fibrinogen (g/L)	0.003	0.137	0.981	0.997	0.762	1.304
D-Dimer (mg/L)	0.836	0.467	0.074	2.307	0.923	5.765
C-reactive protein (mg/L)	0.020	0.010	0.040^∗^	1.02	1.001	1.040
Serum ferritin (*μ*g/L)	0.000	0.001	0.788	1.00	0.999	1.001
LDH (units/L)	0.004	0.004	0.389	0.996	0.988	1.005

^∗^ *p* value < 0.05.

## Data Availability

Our institution restricts access to data due to patient confidentiality, ethical concern, and institution ownership.
